# Surgical aspects in craniopharyngioma treatment

**DOI:** 10.1515/iss-2019-1004

**Published:** 2020-10-30

**Authors:** Shingo Fujio, Tomoko Hanada, Masanori Yonenaga, Yushi Nagano, Mika Habu, Kazunori Arita, Koji Yoshimoto

**Affiliations:** Department of Neurosurgery, Graduate School of Medical and Dental Sciences, Kagoshima University, Kagoshima, Japan; Pituitary Disorders Center, Kagoshima University Hospital, Kagoshima, Japan; Department of Neurosurgery, Izumi regional medical center, Akune, Japan

**Keywords:** craniopharyngioma, Gamma Knife, pituitary function, surgery

## Abstract

**Objectives:**

Total surgical resection is the gold standard in the treatment of craniopharyngioma. However, there is concern that aggressive surgical resection might result in high rates of endocrinologic, metabolic, and behavioral morbidities. Subtotal resection (SR) with subsequent radiation therapy (RT) may reduce surgical complications, but it may also increase the risk of tumor recurrence and radiation-induced side effects. Therefore, the optimal surgical strategy remains debatable.

**Methods:**

To determine the optimal surgical strategy, we assessed the clinical courses of 39 patients (19 male patients and 20 female patients) with newly diagnosed craniopharyngioma who were treated at our institute. The median age at diagnosis was 34 years (range: 0–76 years). The median follow-up period was 8.5 years (range: 3–160 months). Our treatment strategy comprised gross total resection (GTR) for craniopharyngioma in patients that were not at surgical risk. Conversely, after adequate tumor decompression, we used RT, mainly Gamma Knife radiosurgery, in patients at risk. We divided the patients into the following three groups depending on the treatment course: GTR, SR with RT, and SR with staged surgery. We compared tumor characteristics, as well as patients’ conditions at the preoperative stage and last follow-up, among the three groups.

**Results:**

There were 8, 21, and 10 patients in the GTR, SR with RT, and SR with staged surgery groups, respectively. There were no differences in the maximum tumor diameter, tumor volume, composition, and presence of calcification among the groups. Among the 39 patients, 24 underwent transcranial microsurgery and 15 underwent trans-sphenoidal surgery as the initial treatment. No cases involving surgical mortality, cerebrospinal fluid leakage, severely deteriorated visual function, or severe hypothalamic damage were observed. No tumor recurrence was noted in the GTR group. One patient required additional RT, and one patient underwent second surgery for tumor recurrence in the SR with RT group. In the SR with staged surgery group, 8 of the 10 patients eventually underwent RT, but tumor control was achieved in all patients at the latest follow-up. In this group, the third trans-sphenoidal surgery caused a severe vascular injury in one patient. At the final follow-up, 33 (85%) patients were undergoing anterior pituitary hormone replacement, and the rate of diabetes insipidus was 51%. There was no significant difference in the pituitary dysfunction rate among the groups.

**Conclusions:**

We observed a low rate of surgical complications and a sufficient tumor control rate in response to our treatment strategy. Despite attempting preservation of the pituitary stalk, we found it difficult to rescue anterior pituitary function.

## Introduction

Despite the advances in medical imaging diagnosis, operative techniques, and surgical equipment, craniopharyngioma is considered a difficult lesion to treat. The optimal treatment strategy for craniopharyngioma remains unclear. When applicable, since craniopharyngioma is a benign tumor, gross-total resection (GTR) is the gold standard of treatment [1], [2], [3]; however, craniopharyngioma surgery remains challenging because of the tumor’s anatomical location and its relationship with surrounding delicate structures, including the optic chiasm, hypothalamus, as well as the internal carotid arteries and their branches [2], [4], [5]. Previous studies have reported that subtotal resection (SR) with subsequent radiation therapy (RT) may reduce surgery complications [6], [7], [8]. However, there are concerns regarding the risk of tumor recurrence and radiation aftereffects [9], [10], [11].

For more than 10 years, to avoid visual impairments and hypothalamic dysfunction in patients with surgical morbidity, we have used RT instead of attempting total tumor removal. In addition, we have also attempted to preserve the pituitary stalk to the extent possible.

In this study, we aimed to review the treatment outcome observed in our institution and discuss future therapeutic strategies for craniopharyngioma.

## Materials and methods

### Patient selection

Between 2006 and 2020, 41 patients with craniopharyngioma underwent 58 operations in Kagoshima University Hospital. We retrospectively reviewed 39 patients with newly diagnosed craniopharyngioma who were treated with transcranial microsurgery (TCM) or trans-sphenoidal surgery (TSS).

### Treatment strategy

Our treatment principle is that patients should be treated without causing any visual impairments or hypothalamic dysfunction. We, therefore, perform complete resection only when the risk of complications is low. For cases involving a risk of surgical complications by total resection, we perform partial resection followed by stereotactic RT. When the size of the residual tumor was deemed too large for stereotactic RT, we apply a staged surgery in which the approach side is altered compared to that of the first surgery. If cyst walls extensively remain, we perform conventional local irradiation. Regarding the pituitary stalk, we attempt to preserve it as much as possible.

### Surgical procedures

Regarding TCM, we used interhemispheric, pterional, transcortical, and orbitozygomatic approach in accordance with the tumor characteristic. Until 2011, TSS was mainly performed using a microscopic view, aided by endoscopic observation. In 2011, we introduced a high-definition (HD) endoscope (Karl Storz SE & Co. KG, Tuttlingen, Germany) and subsequently initiated fully endoscopic endonasal surgery. We fixed this endoscope with a UNIARM (Mitaka Kohki Co., Ltd., Tokyo, Japan). We have used the bi-nostril approach since 2012. If possible, a lumbar drainage was inserted preoperatively and removed on the third day after surgery.

### Neuroimaging analysis

We conducted preoperative magnetic resonance imaging (MRI) to determine the tumor size, volume, composition, and location. The tumor size was measured as the maximum measurable diameter on MRI. The tumor volume was calculated using the ABC/2 formula. Tumor composition was classified as either solid or cystic; specifically, if the solid component represented >50% of the volume, the tumor was defined as solid, and if not, it was defined as cystic. Bone computed tomography images were evaluated for intratumoral calcification presence. Based on their origin and growth pattern, we defined craniopharyngioma as follows: the intrasellar, prechiasmatic, retrochiasmatic, and intra-third ventricle type, as adapted from a study by Morisako et al. [3].

### Endocrinological status

We defined anterior pituitary hormone dysfunction as abnormally low basic levels of free thyroxine, thyroid-stimulating hormone, cortisol, adrenocorticotropic hormone, testosterone, estradiol, or insulin-like growth factor I or when there was hormone supplementation use. Patients using desmopressin were considered to have diabetes insipidus (DI).

### Performance status

We assessed functional performance status using the Karnofsky Performance Status (KPS) scale, with functional deficit being considered as either a KPS score <70 or the inability to resume a previous occupation.

### Clinical evaluation of patients depending on treatment strategy

We divided the patients into three groups based on the treatment course as follows: GTR group; SR with RT group; and SR with staged surgery group. We compared the tumor characteristics, as well as the patients’ conditions at the preoperative stage and last follow-up, among the three groups.

### Statistical analysis

All statistical analyses were performed using Starflex software (version 6.0; Artech Co. Ltd., Osaka, Japan). Based on the dataset characteristics, the data were analyzed using Mann–Whitney’s U test, as well as Fisher’s exact test. Differences with a p-value of <0.05 were considered statistically significant.

## Results

Table 1 summarizes the patients’ characteristics, tumor characteristics, and conditions before surgery and at the last follow-up. There were 8, 21, and 10 patients in the GTR group, SR with RT group, and SR with staged surgery group, respectively. The median tumor removal rate in the SR with RT and SR with staged surgery groups was 98%.

**Table 1:** Patient characteristics, tumor characteristics, and conditions before surgery and at the last follow-up.

	All patients	GTR group	p-Value (GTR vs. SR + RT)	SR with RT group	p-Value (SR + RT vs. Staged surgery)	SR with staged surgery group	p-Value (Staged surgery vs. GTR)
Number of patients	39	8	n/a	21	n/a	10	n/a
Median age (years)	34 (0–76)	36.5 (8–55)	NS	19.0 (0–76)	NS	49 (3–76)	NS
Sex (M/F)	19/20	4/4	NS	11/10	NS	4/6	NS
**Tumor characteristics**							
Maximum diameter (mm)	28 (12–96)	24 (12–60)	NS	28 (20–96)	NS	28 (20–49)	NS
Tumor volume (cc)	7.2 (0.6–376.0)	3.8 (0.6–15.5)	NS	7.1 (2.6–376.0)	NS	7.1 (2.4–25.1)	NS
Composition (Solid/Cystic)	10/29	3/5	NS	3/18	NS	4/6	NS
Calcification (Yes/No)	26/13	5/3	NS	16/5	NS	5/5	NS
Anatomical sub-classification (Intrasellar/prechiasmatic/retrochiasmatic/Intra-third-ventricle)	7/13/12/7	3/0/3/2	n/a	3/9/4/5	n/a	1/4/5/0	n/a
**Patients’ preoperative condition**							
KPS scores	90 (40–100)	90 (70–100)	NS	90 (50–100)	NS	90 (40–100)	NS
Physical or mental disability (Yes/No)	8/31	1/7	NS	5/16	NS	2/8	NS
Hypopituitarism (Yes/No)	24/15	6/2	NS	11/10	NS	7/3	NS
Diabetes insipidus (Yes/No)	3/36	1/7	NS	0/21	NS	2/8	NS
**Surgical procedures**							
Transcranial microsurgery/Trans-sphenoidal surgery	24/15	2/6	NS	14/7	NS	8/2	NS
**Patients’ condition at the last follow-up**							
KPS scores	90 (40–100)	100 (90–100)	NS	100 (60–100)	0.01	90 (40–100)	NS
Physical or mental disability (Yes/No)*	4/32	0/8	NS	2/18	NS	2/6	NS
Hypopituitarism (Yes/No)	33/6	6/2	NS	17/4	NS	10/0	NS
Diabetes insipidus (Yes/No)	20/19	5/3	NS	9/12	NS	6/4	NS

*We excluded three cases. One patient died of unexplained chemical meningitis, the patriarch patient died of urinary bladder cancer, and the other patient had subarachnoid hemorrhage due to an aneurysm at a location unrelated to the tumor.

Data are presented as median (range) unless otherwise specified. GTR, gross-total resection; SR, subtotal resection; RT, radiation therapy; KPS, Karnofsky Performance Status; n/a, not applicable; NS, not significant.

### Patient characteristics

Among the patients, there were 19 male and 20 female patients. The median age at diagnosis was 34 years (range: 0–76 years). There were 14 pediatric patients (35.9%), i.e., patients aged ≤18 years. The median follow-up duration was 8.5 years (range: 3–160 months). There was no among-group difference in the median age and sex ratio.

### Tumor characteristics

There were no significant among-group differences in the maximum tumor diameter, tumor volumes and composition, and calcification presence. There were fewer cases of intrasellar and intra-third ventricle types compared with those of the pre- and retro-chiasmatic type, but three of the eight patients in the GTR group exhibited the intrasellar type.

### Surgical results

Among the 39 patients, 24 underwent TCM, while 15 underwent TSS. Regarding TCM, 11, 10, 2, and 1 were resected using an interhemispheric, pterional, transcortical, and orbitozygomatic approach, respectively. Although TCM was the main surgical approach in SR with RT group and SR with staged surgery group, six of eight cases in the GTR group were selected TSS. There was an increase in the TSS use frequency after introducing the HD endoscope. In the last three years, five of seven (71%) patients had undergone tumor removal through TSS. Lumbar drainage was used for 8 of 15 (53%) patients. The pituitary stalk was morphologically preserved during surgery in 35 of the 39 (90%) patients.

### Postoperative complications

There was no case of surgical mortality, cerebrospinal fluid (CSF) leakage, or deteriorated visual function. Moreover, there was no new case with symptoms of severe hypothalamic dysfunction, such as central fever, uncontrollable overeating, or adipsia. In the GTR group, there was no surgical morbidity. In the SR with RT group, we confirmed abducens nerve palsy in one patient; however, the patient completely recovered within one month. Another patient had subarachnoid hemorrhage, which resolved spontaneously without complications. In the SR with staged surgery group, one patient experienced temporary psychotic disorders; however, he was able to return to work after rehabilitation. Another patient required drainage for a chronic subdural hematoma. Moreover, among the patients that underwent repeated TSS, there were two cases of vascular injury that occurred during the second and third operations. Among these two cases, one was asymptomatic, while the other was rendered bedridden.

### Radiotherapy

In the SR with RT group, 17 patients underwent Gamma Knife radiosurgery (GKRS) in a single session. The prescription dose to the tumor margin was 14 or 15 Gy. One patient underwent fractionated GKRS with the prescription dose of each fraction being 4.5 Gy. Two patients underwent a combination of conventional local irradiation and GKRS (30 Gy and 40 Gy + GKRS 8 Gy). Another patient underwent intensity-modulated radiotherapy with 54 Gy.

### Postoperative course

**Figure 1:**
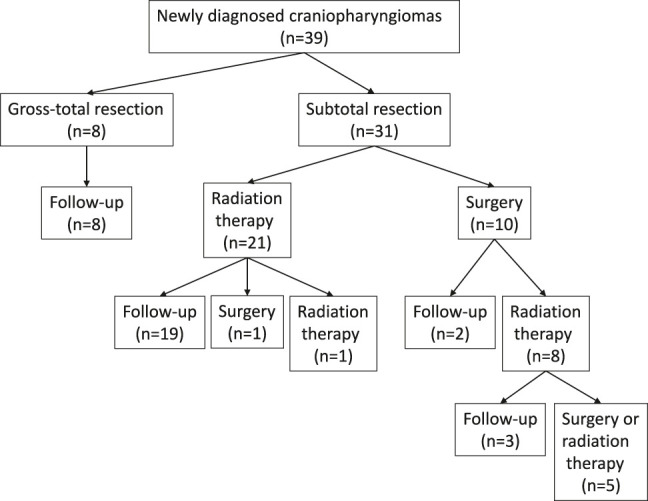
Postoperative course of 39 craniopharyngioma patients.

The postoperative course is depicted in Figure 1. There was no tumor recurrence in the GTR group. In the SR with RT group, one patient required additional GKRS for tumor recurrence. Another patient underwent a second surgery for a recurrent tumor that occurred out of the irradiation field. In the SR with staged surgery group, 8 of 10 (80%) patients eventually had to undergo RT (7 GKRS, 1 cyberknife). Among those patients, five required repeat RT or additional surgery. Nonetheless, tumor control was achieved in all patients at the last follow-up. One died of unexplained chemical meningitis 12 years after her first operation. The patriarch patient died of urinary bladder cancer two years after surgery. Another patient had subarachnoid hemorrhage due to an aneurysm in a location unrelated to the tumor. We excluded these cases in the evaluation of the postoperative performance state.

### Endocrinological outcome

Regarding the preoperative status, 24 of the 39 (62%) patients had impaired anterior pituitary function, while 3 (8%) had DI. At the time of final follow-up, 33 (85%) patients were receiving anterior pituitary hormone replacement, while 20 (51%) patients had DI. There was no significant among-group difference in the pituitary dysfunction rates.

### Performance status

Preoperatively, 8 of the 39 (21%) patients experience physical or mental disability. At the last follow-up, the KPS score improved in 20 patients, remained stable in 13 patients, and deteriorated in three patients, with two of the latter patients being in the SR with staged surgery group. The postoperative KPS scores in the SR with staged surgery group were significantly lower than those in the SR with RT group. All pediatric patients, except one who had myopathy, were able to enter regular class.

### Representative cases

#### Case 1

**Figure 2:**
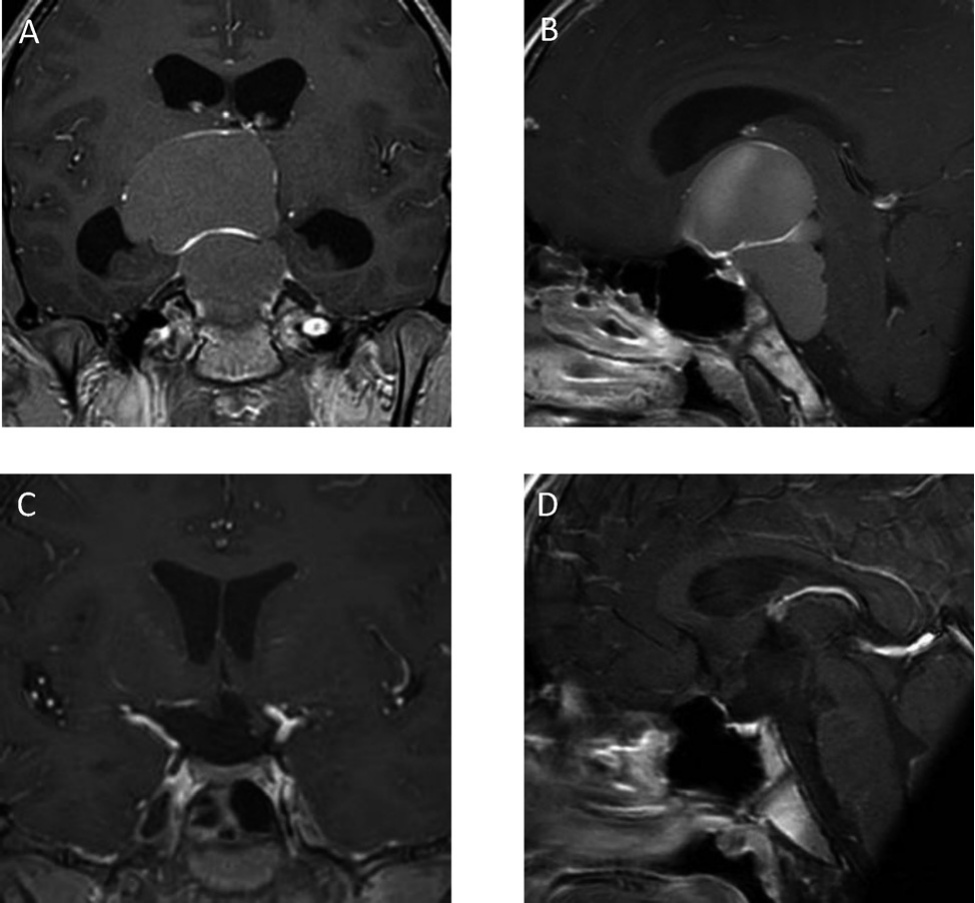
Case 1: A 13-year-old boy. Preoperative coronal (A) and sagittal (B) enhanced T1-weighted magnetic resonance imaging (MRI) demonstrated an expanded cystic tumor that encroached the third ventricle and posterior circulation with hydrocephalus. The tumor was totally removed, with postoperative MRI showing no residual tumor (C, D).

A 13-year-old boy presented to our hospital with hemianopsia on the left side and increased intracranial pressure. MRI showed an expanded tumor that encroached the third ventricle and posterior circulation with hydrocephalus (Figure 2A, B). The tumor was totally removed via the orbitozygomatic approach (Figure 2C, D). Postoperatively, he was able to continue his school life with hormone replacement therapy.

#### Case 2

**Figure 3:**
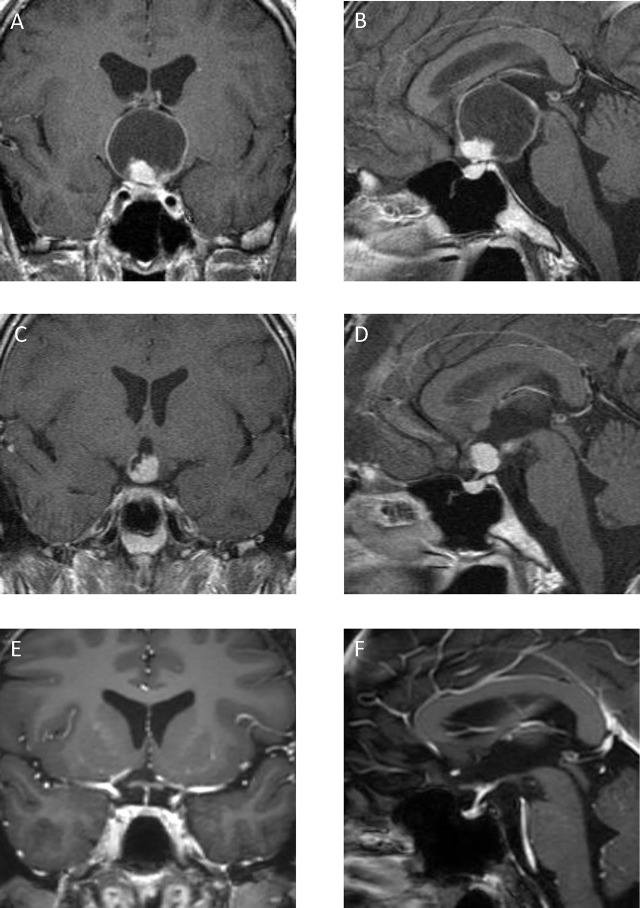
Case 2: An 18-year-old man. Preoperative coronal (A) and sagittal (B) enhanced T1-weighted magnetic resonance imaging (MRI) showed a mainly cystic tumor, which extended to the third ventricle. Postoperative enhanced T1-weighted MRI showing a residual tumor in the third ventricle (C, D). There was no tumor recurrence after performing a combination of conventional local irradiation and Gamma Knife radiosurgery (E, F).

An 18-year-old man presented with visual deterioration and an occasional headache. Preoperative MRI showed a mainly cystic tumor extending to the third ventricle (Figure 3A, B). Tumor removal was performed via the interhemispheric translaminaterminalis approach. The tumor was strongly adherent to the hypothalamus and optic chiasm, and surgery was completed through partial excision (Figure 3C, D). We performed a combination of 40 Gy conventional local irradiation and 8 Gy GKRS. Since then, 10 years have passed, and he does not require hormone replacement; further, he is now a father without an episode of tumor recurrence (Figure 3E, F).

#### Case 3

**Figure 4:**
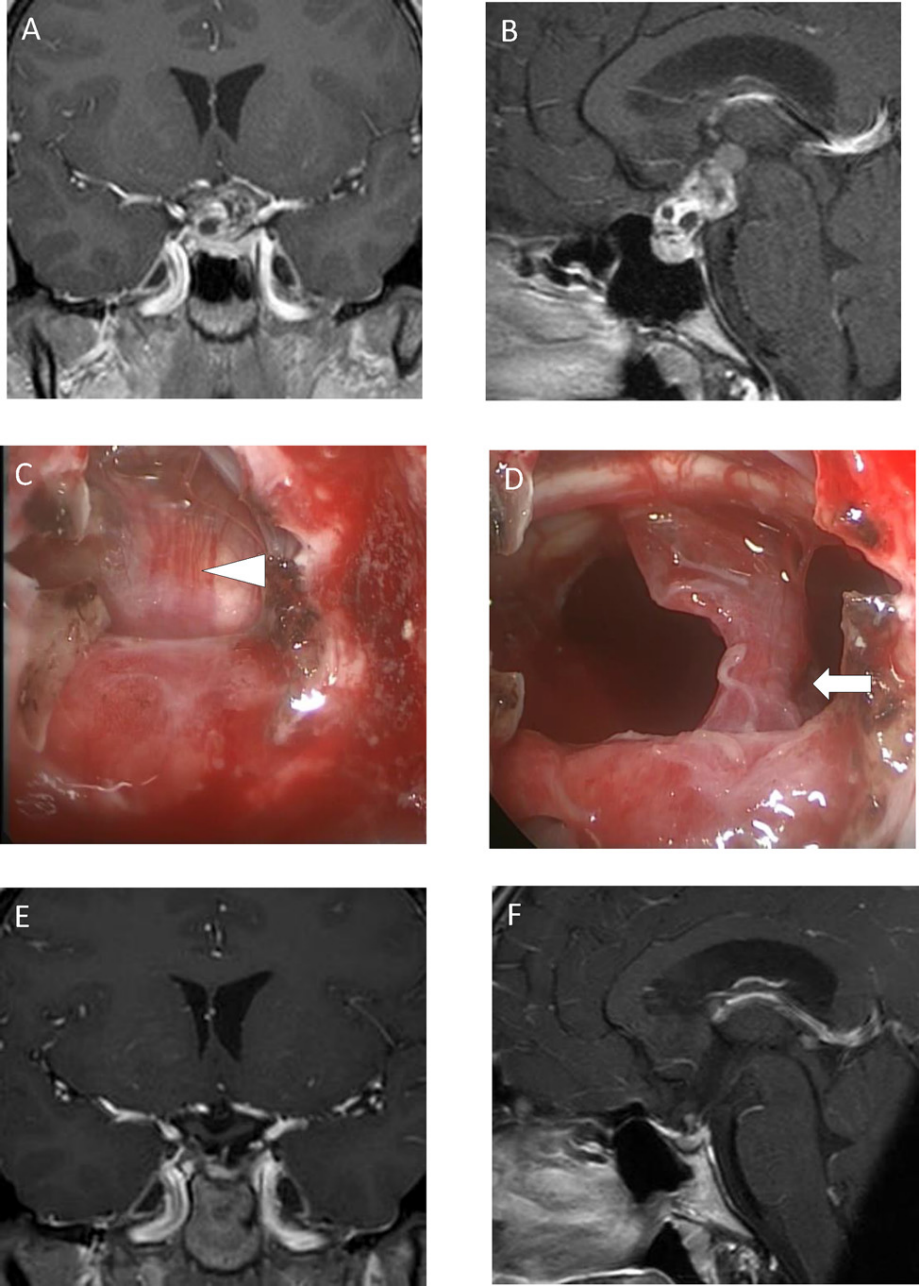
Case 3: A 14-year-old girl. Preoperative coronal (A) and sagittal (B) enhanced T1-weighted magnetic resonance imaging demonstrated a solid suprasellar tumor. The intraoperative view showed that the pituitary stalk was located rostral to the tumor (C). The arrow head points to the pituitary stalk. The tumor was removed except for the pituitary stalk and the tumor caudal to it (D). The arrow points to the residual tumor caudal to the pituitary stalk. After Gamma Knife radiosurgery, the tumor was completely controlled (E, F).

A solid suprasellar tumor was detected in a 14-year-old girl after she hit her head (Figure 4A, B). Endoscopic endonasal surgery was performed, and the tumor was located behind the pituitary stalk (Figure 4C). We removed tumors that were not attached to the pituitary stalk (Figure 4D). The pituitary stalk was preserved; however, she lost pituitary function immediately after surgery. After 15 Gy GKRS, the tumor was completely controlled (Figure 4E, F). She still requires hormone replacement therapy; however, she attends school without difficulty.

## Discussion

### Transcranial microsurgery vs. trans-sphenoidal surgery

Historically, TCM had been the main approach for reaching and removing craniopharyngioma [12]; this technique includes the pterional and orbitozygomatic, subfrontal, interhemispheric translaminaterminalis, and transcallosal–transventricular approaches [2], [3], [13], [14], [15]. A successful surgical outcome can be achieved through anatomical consideration of craniopharyngioma based on the tumor origin and growth pattern [3]. However, TCM approaches present a risk of brain retraction, and it is difficult to observe the lower surface of the chiasm. On the contrary, TSS, especially endoscopic endonasal approaches, provides an excellent view of the operative field without brain retraction and manipulation [16]. Over the last decade, endoscopic endonasal approaches have become an important surgical method for treating craniopharyngioma [1], [15], [16]. Indeed, there was also an increase in the TSS use frequency in our institution.

The superiority of TCM vs. TSS remains controversial [2], [5], [13], [15]. In 2012, Komotar et al. reviewed 3,470 patients with craniopharyngioma to assess open and endoscopic surgical series [13]. Compared with the transcranial group, the endoscopic group underwent a significantly higher number of GTRs, and they also exhibited a significantly greater improved visual activity. Moreover, Mende et al. reviewed 148 patients with adult-onset craniopharyngioma [5] and found a significantly higher electrolyte imbalance frequency after transcranial approaches than after TSS. Moreover, they reported that patients who had undergone transcranial surgery presented significantly higher rates of pituitary dysfunction than did patients who had undergone TSS. However, another study reported no significant between-group difference in the postoperative pituitary dysfunction rate [15]. TSS has a higher risk of CSF leakage compared to that of TCM [12]. The CSF leakage incidence in TSS for craniopharyngioma has been reported to be approximately 10% [1], [2], [15]; however, the frequency decreases with surgeon experience [1], [16]. Recent studies have demonstrated the efficacy of multilayer reconstruction and the dural suturing technique in preventing CSF leakage [17], [18].

We did not experience a case of CSF leakage in patients who underwent TSS. Our method of sellar reconstruction was simple. We used a fat graft and covered it with fibrin glue-soaked gelatin sponge (FGGS). The sellar floor was reconstructed with a vomer splint and covered again with FGGS. We have previously presented the details of this method [19].

Indications for endoscopic surgery for craniopharyngioma may expand in the future. From a retrospective perspective, we believe that some cases that would traditionally be removed using TCM will instead be removed using TSS. However, we consider tumors with excessive lateral extension (such as Case 1) or excessive calcification as poor candidates for endoscopic endonasal surgery.

### Aggressive surgery vs. surgery using RT

Radical resection is considered the most effective treatment for craniopharyngioma with a low recurrence risk [1], [3], [14], [20]. Previous studies that recommended aggressive tumor removal reported that the rates of GTR or near-total resection were between 80 and 90%, while the local recurrence rate was around 10% [1], [2], [3], [14]. However, there is concern that aggressive surgical resection could lead to high rates of endocrinologic and behavioral morbidity. Therefore, SR with subsequent RT has been considered an acceptable treatment strategy for craniopharyngioma. A review of 442 patients presented no significant difference in progression-free survival and overall survival between the GTR group and SR + RT group [21]. Sughrue et al. conducted a literature review on morbidity resulting from craniopharyngioma treatment [4] and reported no significant difference in the neurologic deficit rates between patients undergoing GTR alone, SR alone, or SR followed by RT. However, patients undergoing GTR had a high rate of developing at least one pituitary hormone deficiency compared to those undergoing SR alone or SR + RT. In contrast, another study reported no significant difference in the rate of anterior pituitary hormone deficiency and DI among patients undergoing GTR, SR alone, or SR + RT [9]. In our cohort, there was no significant among-treatment strategy difference in the pituitary dysfunction rates at the last follow-up.

We preserved the pituitary stalk in 90% of the patients; however, the rate of anterior pituitary dysfunction was 85%, which is similar to that in previous reports that recommended aggressive tumor removal [1], [3], [14]. Contrastingly, compared to our cohort (51%), the rate of postoperative DI was higher (70–90%) in those previous reports [1], [3], [14]. This indicates that preserving the pituitary stalk may not maintain anterior pituitary function but may help reduce DI incidence.

At the last follow-up, KPS scores in the SR with staged surgery group were lower than those in the SR with RT group. Specifically, the SR with staged surgery group had many intractable cases with repeated recurrences. Moreover, two vascular injuries occurred during the reoperations. It is a limitation of the present study that we had some cases in which reoperation was only required because GKRS was not initiated within the ideal timeframe of the therapeutic intervention. Reoperation is accompanied by a high risk of complications [1]; therefore, surgeons should avoid unnecessary reoperation as much as possible. For this purpose, frequent imaging follow-ups are important for patients with residual lesions.

### Future treatments

GKRS for craniopharyngioma has been reported to be effective. In our institution, the tumor control rate by GKRS was 76%, which is similar to that of previous reports [22], [23], [24]. However, one of the crucial complications of GKRS is optic nerve injury [23], [24]; therefore, we could not apply GKRS for tumors in contact with the optic pathway. There has been a recent acceptance of the use of fractionated GKRS for perioptic tumors [24], [25], which was found to be as effective as single session GKRS with a low risk of optic pathway damage [24], [25]. Therefore, it might be possible to treat perioptic craniopharyngioma with fractionated GKRS.

Considering that a high rate of papillary craniopharyngioma exhibits a BRAF V600E mutation [26], [27], the efficacy of the targeted therapy has been widely reported [28], [29], [30]. Juratli et al. reported the first example of successful neoadjuvant treatment in a patient with BRAF V600E mutant craniopharyngioma after tumor biopsy [31]. Preoperative administration of BRAF inhibitors is expected to shrink the tumor and prevent surgery-associated complications. Noninvasive molecular diagnosis of craniopharyngioma may be possible using diagnostic imaging [32], [33] or liquid biopsy [28]. In the future, targeted therapy may change the role of surgery.

## Conclusion

We have attempted GTR for craniopharyngioma in patients not at surgical risk, and used RT, mainly GKRS, after adequate tumor decompression in patients at risk. This avoided the risk of severe hypothalamic dysfunction, visual disturbance, and surgical mortality, with good tumor control. We found it difficult to rescue anterior pituitary function, even after actively preserving the pituitary stalk; nevertheless, this approach may help reduce DI incidence. Considering the availability of new treatment options, including fractionated GKRS and medication therapies, it is necessary to conduct long-term studies on the optimal surgical strategies.
