# Supplementary material for: Surgical aspects in craniopharyngioma treatment
Source: Innov Surg Sci. 2020 Oct 30;6(1):25–33. doi: 10.1515/iss-2019-1004 (PMC8668032; doi:10.1515/iss-2019-1004)
Supplement: Supplementary file 1 [file iss-06-20191004-s001.pdf]

Reviewer Assessment

Shingo Fujio: Surgical aspects in craniopharyngioma treatment

Reviewers' Comments to Original Submission

Reviewer 1: anonymous

Date received: 11-Jul-2020
Reviewer recommendation: **Return to author for minor modifications**
Reviewer overall scoring: **High**

Assessment Form scores: 5 = High/Yes; 3 = Medium/Adequate; 1 = Low

Is the subject area appropriate for the journal	5				
Does the title clearly reflect the paper's content?	5				
Does the abstract clearly reflect the paper's content			3		
Do the keywords clearly reflect the paper's content?			3		
Does the introduction present the problem clearly?	5				
Are the results/ conclusions justified?	5				
How comprehensive and up-to-date is the subject matter presented?		4			
How adequate is the data presentation?	5				
Are units and terminology used correctly?			3		
Is the number of cases adequate?	5				
Are the experimental methods/ clinical studies adequate?	5				
Is the length appropriate in relation to the content?	5				
Does the reader get new insights from the article?		4			
Please rate the practical significance.	5				
Please rate the accuracy of methods.	5				
Please rate the statistical evaluation and quality control.	5				
Please rate the appropriateness of the figures and tables.	5				
Please rate the appropriateness of the references.		4			
Please evaluate the writing style and use of language.	5				
Please judge the overall scientific quality of the manuscript.		4			
Are the methods used worthy of reproduction in greater deal?	No response				
Would you be willing to review a revision of this manuscript?	Yes				

Comments to author: Since many years, there is a discussion ongoing, how to treat craniopharyngiomas. The major concern is the preservation of the pituitary function and the avoidance of hypothalamic-hypophyseous dysfunctions. Therefore, the topic of the paper is relevant and actual.
There are only minor shortcomings in the paper that should be corrected:

1. The abstract is too long. It should concentrate in a brief manner on: the goal of the study, the method, the results, the conclusion.
2. Keywords: BRAF has to be taken out. This issue has not been discussed in the paper - no data. BRAF will be an important parameter for future treatment strategies, which has been briefly mentioned under future perspectives.
3. Concerning the endocrinological status: The diagnosis of diabetes insipidus (DI) basis on sodium, osmolality and specific weight of the urine and serum. The term polyuria by itself is not enough for the diagnosis DI. Please, correct.
4. The Karnofsky score (KPS) stands for (in)dependence in the daily living and quality of life. The term "neuropsychological workup/ status" is misleading and should be corrected. The conclusions are sound. The literature does not include the last two years (2019, 2020). Please, check and complete.

Reviewer 2: anonymous

Date received: 13-Jul-2020
Reviewer recommendation: **Return to author for minor modifications**
Reviewer overall scoring: **High**

Assessment Form scores: 5 = High/Yes; 3 = Medium/Adequate; 1 = Low

Is the subject area appropriate for the journal		4			
Does the title clearly reflect the paper's content?	5				
Does the abstract clearly reflect the paper's content		4			
Do the keywords clearly reflect the paper's content?		4			
Does the introduction present the problem clearly?		4			
Are the results/ conclusions justified?		4			
How comprehensive and up-to-date is the subject matter presented?		4			
How adequate is the data presentation?		4			
Are units and terminology used correctly?	5				
Is the number of cases adequate?		4			
Are the experimental methods/ clinical studies adequate?		4			
Is the length appropriate in relation to the content?		4			
Does the reader get new insights from the article?		4			
Please rate the practical significance.		4			
Please rate the accuracy of methods.		4			
Please rate the statistical evaluation and quality control.		4			
Please rate the appropriateness of the figures and tables.		4			
Please rate the appropriateness of the references.		4			
Please evaluate the writing style and use of language.		4			
Please judge the overall scientific quality of the manuscript.		4			
Are the methods used worthy of reproduction in greater deal?	Yes/No				
Would you be willing to review a revision of this manuscript?	Yes/No				

Comments to author: The topic is of high interest and the manuscript is well-written. There are some minor issues / questions:
- The rate of 0% of cerebrospinal fluid leakage is remarkable. Did you use lumbar drainage in patients?
- No data on visual impairment in patients are reported. Please provide data on this issue and also the effects of surgery / radiotherapy on visual function.

- You report “after adequate tumor decompression” in the abstract section. Have you got data on the volume of remnant tumor in cases of incomplete resection?
- Two patients suffered vascular injury. Were these during transcranial or transsphenoidal surgery?
- Olfactory function has a major impact on the quality of life in these patients. Have you got data on olfactory function? This would be of high interest, especially in light of the ongoing discussion regarding advantages of endoscopic techniques.

Authors’ Response to Reviewer Comments

Date received: DD-Mon-2020

Response to reviewer 1

1 The abstract is too long. It should concentrate in a brief manner on: the goal of the study, the method, the results, the conclusion.
Response:
Thank you for your comment. The abstract has been edited with word count reduction in mind.

2 Keywords: BRAF has to be taken out. This issue has not been discussed in the paper - no data. BRAF will be an important parameter for future treatment strategies, which has been briefly mentioned under future perspectives.
Response:
I have removed BRAF from the Keywords.

3 Concerning the endocrinological status: The diagnosis of diabetes insipidus (DI) basis on sodium, osmolality and specific weight of the urine and serum. The term polyuria by itself is not enough for the diagnosis DI. Please, correct.
Response:
Only cases using desmopressin were redefined as DI. We changed the following sentences in the Materials and Methods section of the revised manuscript:
“Patients using desmopressin were considered to have diabetes insipidus (DI).”
The number of preoperative DI changed from 4 to 3 (Table 1)

4 The Karnofsky score (KPS) stands for (in)dependence in the daily living and quality of life. The term "neuropsychological workup/ status" is misleading and should be corrected.
Response:
We changed the neuropsychological workup/status to performance status.

The conclusions are sound. The literature does not include the last two years (2019, 2020). Please, check and complete.
Response:
We submitted this manuscript in April 2020. We included all the surgical cases until April 2020. We changed the following sentences in the Materials and Methods section in the revised manuscript:
Between 2006 and 2020, 41 patients with craniopharyngioma underwent 58 operations in Kagoshima University Hospital.

Response to reviewer 2

1. The rate of 0% of cerebrospinal fluid leakage is remarkable. Did you use lumbar drainage in patients?
Response:
Thank you for your comment. We attempted to use lumbar drainage as much as possible.

Eight of 15 (53%) patients who underwent TSS used lumbar drainage. We changed the following sentences in the Materials and Methods and results section in the revised manuscript:
"If possible, a lumbar drainage was inserted preoperatively and removed on the third day after surgery."
Lumbar drainage was used for 8 of 15 (53%) patients.

2. No data on visual impairment in patients are reported. Please provide data on this issue and also the effects of surgery / radiotherapy on visual function.
Response:
Only about half of the patients had undergone detailed examination for visual function such as static visual field examination because there are some patients with disorientation and pediatric cases. Since other cases were evaluated using information from their family members or simple tests such as the confrontation test, we only conducted a simple evaluation of whether there is a serious visual field disorder or not.

3. You report "after adequate tumor decompression" in the abstract section. Have you got data on the volume of remnant tumor in cases of incomplete resection?
Response:
We added the following sentences in the Results section of the revised manuscript:
"The median tumor removal rate in the SR with RT and SR with staged surgery groups was 98%."

4. Two patients suffered vascular injury. Were these during transcranial or transsphenoidal surgery?
Response:
These two vascular injuries occurred during the repeated TSS. We changed the following sentences in the Results section in the revised manuscript:
"Moreover, among the patients that underwent repeated TSS, there were two cases of vascular injury that occurred during the second and third operations."

5. Olfactory function has a major impact on the quality of life in these patients. Have you got data on olfactory function? This would be of high interest, especially in light of the ongoing discussion regarding advantages of endoscopic techniques.
Response:
Thank you for your comment, but we do not have enough data about olfactory function. We intend to investigate them in the future.

Reviewers' Comments to Revised Submission

Reviewer 2: anonymous

Date received: 29-Sep-2020
Reviewer recommendation: **Accept in present form**
Reviewer overall scoring: **High**

Assessment Form scores: 5 = High/Yes; 3 = Medium/Adequate; 1 = Low

Is the subject area appropriate for the journal		4			
Does the title clearly reflect the paper's content?		4			
Does the abstract clearly reflect the paper's content		4			
Do the keywords clearly reflect the paper's content?	5				

Does the introduction present the problem clearly?	5				
Are the results/ conclusions justified?					
How comprehensive and up-to-date is the subject matter presented?		4			
How adequate is the data presentation?	5				
Are units and terminology used correctly?		4			
Is the number of cases adequate?		4			
Are the experimental methods/ clinical studies adequate?		4			
Is the length appropriate in relation to the content?		4			
Does the reader get new insights from the article?		4			
Please rate the practical significance.		4			
Please rate the accuracy of methods.		4			
Please rate the statistical evaluation and quality control.		4			
Please rate the appropriateness of the figures and tables.		4			
Please rate the appropriateness of the references.		4			
Please evaluate the writing style and use of language.		4			
Please judge the overall scientific quality of the manuscript.		4			
Are the methods used worthy of reproduction in greater deal?	No				
Would you be willing to review a revision of this manuscript?	Yes				

Comments to author: All concerns have been adressed adequately.

Comments by the Editor-in-Chief to Revised Submission

All reviewer concerns raised were addressed satisfactorily. The manuscript may be published in the present stage.
